# Poly(ethylene terephthalate) Powder—A Versatile Material for Additive Manufacturing

**DOI:** 10.3390/polym11122041

**Published:** 2019-12-09

**Authors:** Hao Gu, Fayez AlFayez, Toseef Ahmed, Zahir Bashir

**Affiliations:** 1 SABIC Global Application Technology Europe, Specialties, Plasticslaan 1, 4612PX Bergen op Zoom, The Netherlands; 2 SABIC Plastics Application Development Centre, Prince Turki Street 1, P.O. Box 5101, Riyadh 11422, Saudi Arabia; FayezAF@sabic.com; 3 SABIC Technology Centre, P.O. Box 42503, Riyadh 11551, Saudi Arabia; Toseef@sabic.com; 4 Catenated Carbon Consultancy Ltd., 192 Wake Green Road, Birmingham B13 9QE, UK; zbashir2703@gmail.com

**Keywords:** additive manufacturing, laser sintering, PET, plastics powder, powder bed fusion

## Abstract

The 3D printing of articles by the effect of a directed laser beam on a plastic powder is a demanding process, and unlike injection molding, very few polymers work well enough with it. Recently, we reported that poly(ethylene terephthalate) (PET) powder has intrinsically good properties for 3D printing. Basic mechanical properties were shown earlier and it was demonstrated that unfused but heat-exposed PET powder does not degrade quickly allowing good re-use potential. In this work, we conducted a detailed comparison of the mechanical properties of PET and polyamide 12 from different build orientations. PET powders with two different molecular weights were used. With the high molecular weight powder, the processing parameters were optimized, and the printed bars showed little difference between the different orientations, which means there is low anisotropy in mechanical properties of built parts. Based on processing experience of the first powder, the second powder with a lower molecular weight was also very printable and complex parts were made with ease from the initial printing trials; since the process parameters were not optimized then, lower mechanical properties were obtained. While the intrinsic material properties of PET (melting and re-crystallization kinetics) are not the best for injection molding, PET is eminently suitable for powder bed fusion.

## 1. Introduction

Beaman and Deckard established a 3D printing method in the mid 1980s using a directed laser beam on a plastic powder [1,2,3]. This is most often called selective laser sintering (SLS), and is now practiced quite widely as a prototyping method. However, its advancement into a large volume production technology has been hampered by a limited range of materials sold at exorbitant prices, and by technical shortcomings, such as residual porosity. Injection molding is a more tolerant fabrication-process, as most thermoplastics can be processed with it. Additive manufacturing (3D printing) with plastic powders, on the other hand, is a more demanding process, and very few polymer materials perform well enough. Polyamide 12 gave the best performance amongst plastic powders, and it became the material par excellence. However, the combination of high price, few workable powders, and residual porosity in the articles, led to a degree of stagnation for the method, so that it could not grow very far out of prototyping to a manufacturing process.

This paper is about the 3D printing of semi-crystalline PET articles from semi-crystalline PET powder. At the outset, we would like to clarify the mechanism of the formation of the articles from PET powder after exposure to a laser, and thereby use scientifically accurate nomenclature. As mentioned, the most commonly process is referred to as “selective laser sintering” (SLS) or “laser sintering” (LS). However, the sintering of particles does not strictly consist of creating a liquid melt, but involves molecular or atomic diffusion across particle boundaries; thus, creating inter-particle bridges to consolidate the powder. In the earliest days, the amorphous plastic, acrylonitrile butadiene styrene (ABS), was used for building articles via a directed laser beam. In the case of amorphous plastics, selective laser sintering is indeed an accurate enough description. Later, semi-crystalline plastics were also tried. In semi-crystalline polymers, heating the particles above the *T*_g_ is not sufficient to cause flow; for consolidation, the melting point has to be crossed. However, the term SLS came to be used for both amorphous and crystalline polymers. With semi-crystalline plastics, the correct mechanistic description is “selective laser melting (SLM).” However, the term SLM today is associated with metal powders. In line with common usage, we have referred in the past to selective laser sintering of semi-crystalline PET [4,5] despite melting occurring, but henceforth we shall use the term “powder bed fusion.” It is a standard term [6] that is increasingly used by others [7].

As of 2019, available commercial plastic powders for powder bed fusion were polyamides (PA12, PA11, PA6), thermoplastic elastomers, thermoplastic polyurethanes, polystyrene, polypropylene, and poly(ether ether ketone). Filled versions of some of these are also available. PA12 holds 90% market share currently [8].

To escape this limitation, new materials are needed from more common or commodity thermoplastics, which would perform as well or better than PA12. One major obstacle was that the bed temperature of the first generation of the machines was limited to ≈200 °C. A few machines were available for processing plastics powders that melted over 300 °C, but these were very costly. Some machine manufacturers are now starting to produce the equipment operating in the 200–300 °C range. 

In the first reported work on the use of PET powder for powder bed fusion, Bashir et al. showed that PET with a melting point of ≈250 °C printed very well [4]. The processing window was wide (≈52 °C), and the articles could be printed with sharp definition and without curling or cracking. The surface finish was even better than that obtained with printed PA12. In a second work, we showed the PET also had good re-use capability, for unfused but heat-exposed powder [9]. However, in these works, the laser processing parameters and only the very basic mechanical properties of an *xy* orientation build were reported from printed bars. The flexural modulus was 2.49 GPa, the flexural strength was 68 MPa, and the elongation-to-break was unreported. However, the *zx* orientation properties have to be considered when proposing a new material for powder bed fusion.

In this work, the mechanical properties of PET were measured from tensile bars printed in *xy*, *xz*, and *zx* build orientations, and compared with equivalents from PA12. Two PET powders with different molecular weights were tried. The higher molecular weight PET was printed in a laboratory machine, after the optimization of the processing parameters to get the best mechanical properties. The lower molecular weight PET was printed in a laboratory machine and a commercial machine, to establish the versatility of this material for powder bed fusion.

## 2. Materials and Methods 

Two types of PET powders were used, and will be called type 1 and type 2 (T1 and T2). Both types were produced in SABIC (Yanbu, Saudi Arabia), but are not commercially available yet.

The PET powder T1 had an intrinsic viscosity (I.V.) of 1.122 dL/g. The particle size parameters for T1 powder were obtained by laser light scattering (Mastersizer 2000, Malvern, Worcestershire, UK): d(10) 24 µm; d(50) 59 µm; d(90) 125 µm.

The crystallinity, the melting, and crystallization behaviors of the powder were assessed by differential scanning calorimetry (Q2000 DSC, TA Instruments, New Castle, DE, USA) by a heat-cool cycle at 10 °C/min. This provided information on the potential printing window. The T1 powder had a DSC heat of fusion of 50–60 J/g, corresponding to a crystallinity of ≈50%, since according to Balta-Calleja et al., the heat of fusion of 100% PET crystal is 118 J/g [10]. In total, 0.05 wt % of a flow promoter (Aerosil 200 nano silica) was added to improve the powder flow. The PET powder T1 with the flow promoter was pre-dried at 130 °C for 3 h in the oven before placing in the laboratory machine.

A laboratory machine Rig P250U (Fraunhofer, Oberhausen, Germany) was used for the printing with PET powder T1. The machine is a prototype machine on which we completed our previous work [4,9]. It had a dry nitrogen flow kept over the head space of the powder bed to minimize degradation. Compared with the commercial machines, this one had a smaller build area (effective printing area length × width × height was 120 mm × 120 mm × 80 mm). One advantage of this machine for material development was the possibility to work with limited amounts of powder. The second advantage of this machine was its high build-bed temperature capability. It could go up to 250 °C, which is necessary for PET powder. The third advantage was the unblocked operating software. The process parameters could be easily modified for optimization.

The laser processing parameters used with T1 are shown in Table 1. The build bed temperature was kept at 225–228 °C. For a complete evaluation of mechanical properties, the test specimen was built from T1 with different printing orientations. Note that for powder T1 and the laboratory machine, considerable work was done to optimize the processing parameters (shown in Table 1), to get the best mechanical properties.

For benchmarking the properties of PET, PA12 bars were also built in the same way under optimized conditions, in the laboratory machine. Duraform^®^ PA12 powder was used as a reference to compare the performance of PET in powder bed fusion. This commercial PA12 powder had particle size range d(10) 34 µm; d(50) 58 µm; and d(90) 88 µm, and it was not known if a flow agent had been added by the supplier.

PET powder T2 had an I.V. of 0.845 dL/g. From laser light scattering, the powder had a particle size range d(10) 25 µm; d(50) 66 µm; and d(90) 103 µm. The crystallinities of this powder and the printing window were also assessed by DSC by a heat-cool cycle at 10 °C/min. The T2 powder was printed in two machines. Firstly, the same laboratory machine as used for T1 was employed. Like the case with PET powder T1, 0.05 wt % Aerosil 200 (fumed silica, Evonik, Hanau, Germany) was added, and the powder was pre-dried at 130 °C for 3 h pre-drying. The laser parameters for printing powder T2 in the laboratory machine are shown in Table 2.

The laboratory machine allowed a laser travel at a speed of 5 m/s. To test the PET′s performance on a machine capable of commercial speeds, the powder T2 was also printed in a commercial machine (effective printing area length × width × height was 235 mm × 235 mm × 305 mm) from LSS Laser-Sinter-Service GmbH, Dortmund, Germany. This machine offered a scan speed of 12.7 m/s. This time, the PET powder T2 was not pre-dried and no flow agent was added to it. This machine also had a dry nitrogen flow kept over the head space of the powder beds. The processing parameters used for the commercial machine with powder T2 are shown in Table 2 as well.

Note that for powder T2, while conditions were chosen for printing with no curl, no optimization of the processing parameters was done in the laboratory machine nor the commercial machine to maximize the mechanical properties.

Micrographs of the fracture surfaces of tensile bars made with PET powder T2 in the commercial machine were obtained using scanning electron microscopy (SEM Thermo Scientific Quanta 200, Eindhoven, The Netherlands). The SEM was operated at 20 kV. The surfaces were sputter-coated with an Au–Pd thin layer of ≈10 nm prior to SEM examination.

## 3. Results and Discussion

### 3.1. Melting and Crystallization Properties of PET Powder T1, Compared to PA12 Powder

There are three melting characteristics of a powder that have a bearing on the print quality. These are (1) the heat of fusion, (2) the melting width, and (3) the processing window = (melting onset) − (crystallization onset).

Bourell et al. [11] generalized the following considerations for selecting the build bed temperature. For semi-crystalline polymers, they stated that the part bed has to be kept 2–4 °C below the melting peak, to minimize the laser energy needed to fuse the powder. If the part bed temperature is too high, caking of the bed occurs due to self-sintering of the powder that makes the left-over material difficult to re-use, and it also causes poor dimensional accuracy in the part. If the bed temperature is lower than this, there is insufficient consolidation, unmelts are left in the part, there is higher porosity, and premature crystallization can occur, causing warping and curling. 

**Figure 1:**
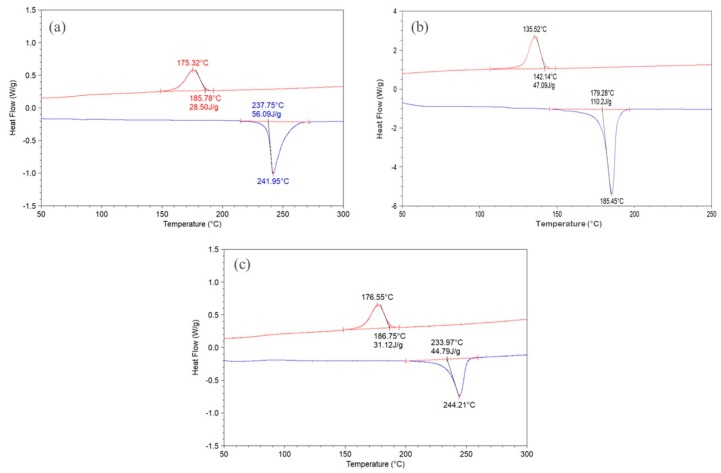
(**a**) DSC of PET powder T1; (**b**) DSC of PA12 powder; (**c**) DSC of printed article from PET T1 shows it is semi-crystalline, with a heat of fusion of 44.8 J/g.

Table 1 shows the optimized laser processing parameters used in the laboratory machine, for PET T1. While for PA12, the build bed temperature is typically held 2–4 °C below the DSC melting peak, with PET T1, we could keep the bed between 225 and 228 °C; that is about 14–17 °C lower than the DSC melting peak (Figure 1a). The heat of fusion of PET is lower than other semi-crystalline polymers, for instance, polyethylene, even at comparable crystallinities. The heat of fusion of PET T1 in Figure 1a was 56.1 J/g and that corresponded to 48% crystallinity [10].

For the PA12 used here, Figure 1b shows the heat of fusion was 110 J/g. For 100% crystalline PA12, Gogolewski et al.′s [12] estimate of the heat of fusion was 209.3 J/g; hence, the PA12 in Figure 1b corresponds to a crystallinity of 53%. Inspection of the literature showed the heat of fusion of commercial PA12 powders disclosed by others was also ≈100 J/g [13]. Thus, for comparable crystallinities of ≈50%, the heat of fusion of the PET will be about half of PA12. As the PA12 powder has about double the heat of fusion of a PET powder, more energy needs to be captured from the moving laser to come to the melting point; hence, the bed has to be kept ≈2–4°C below the peak melting point. Since the heat of fusion of the semi-crystalline PET particles is only about half that of PA12, the bed can be kept 14–17 °C lower than the melting peak. Being able to keep the part bed at a lower temperature decreases energy consumption and helps with minimizing the degradation of the unconverted powder, preserving its re-use capability. Our preference is to keep a lower bed temperature, partly because many current machines are built for PA12 and have a maximum attainable bed temperature of 200–210 °C.

In order to widen the processing window of PA12, Scholten and Christoph [14] aimed to create one that had the largest possible difference between melting and crystallization temperatures, and a melting enthalpy higher than other PA12s. Their PA12 had an enhanced melting point of 185–189 °C, an enthalpy of fusion of 112 ± 17 J/g, and a solidification range of 138–143 °C. They considered that a high heat of fusion to be advantageous as it prevents powder particles located adjacent to the scanned area from the beginning from fusing due to the heat conduction; that is, it reduces lateral growth and this should result in sharper features. High crystallinity leads to two effects: a narrower peak width with a raised melting onset, and a higher heat of fusion. The narrow peak width is good for partial resolution, and the high melting onset widens the process window. However, the higher heat of fusion means higher power is needed and the build bed has to be kept at a higher temperature, which is damaging for re-use of the powder. The broader melting peak would lead to lower print resolution, but we have not found PET to be significantly worse than PA12 powders in this aspect. It is difficult to make a PET powder with a crystallinity much above 50% with the heat of fusion higher than about 60 J/g, and hence, narrowing its melting peak would be difficult. However, the broadness of the melting peak can be reduced to some extent by having a narrower range of particle sizes. Taking the melting width as the melting endpoint in °C—extrapolated melting onset in °C, from the DSC in Figure 1a, PET powder T1 had a melting width of 270 − 238 = 32 °C, while PA12 (Figure 1b) had a melting width of ≈198 − 179 = 19 °C. Perhaps PET′s intermediate heat of fusion of 50–60 J/g, and the melting width of 32 °C versus the PA12′s 19 °C (see Table 3), strike a good compromise between reducing the degradation rate by allowing the build bed to be kept at a lower temperature and obtaining sufficient print resolution.

Further, Bourell et al. [11] noted that small regional temperature variations across the printing bed arise due to irregular heating elements and other factors, and that leads to the differences in quality (porosity) between the parts built in different areas of the building chamber. Referring to the polymers such as PA12, where the build bed has to be kept at 3–4 °C lower than the peak melting temperature, a variation of even 2 °C across the build bed is a major fraction of the 3–4 °C; if there is a colder spot in the bed, there will be curl, while a hot spot would cause melting extending beyond the desired boundaries of the article. In light of this, PET would be more tolerant to temperature variation across the printing bed due to the fact that it does not have to be kept so close to the melting point as with PA12 and other semi-crystalline polymers.

The laser processing window, on the other hand, is related to the separation of the melting and crystallization events and can be taken from the DSC curve as the melting onset temperature − crystallization onset temperature. For PET powder T1, the processing window from Figure 1a is 238 − 186 = 52 °C. 

The processing window of the PA12 used here was estimated from Figure 1b. The melting onset − crystallization onset = 179 − 142 = 37 °C. Scholten and Christoph′s enhanced PA12 had a processing window of 42 °C [14]. This remains lower than the 52 °C for PET powder T1.

In summary, PET powder will have a wider laser processing window, but will have a broader melting width and a lower heat of fusion than PA12 powder. Table 3 collects together the melting width, the heats of fusion and the processing windows of PET powders T1 and T2, and PA12, from their DSC curves.

### 3.2. Mechanical Properties of Articles Printed from PET Powder T1

**Figure 2:**
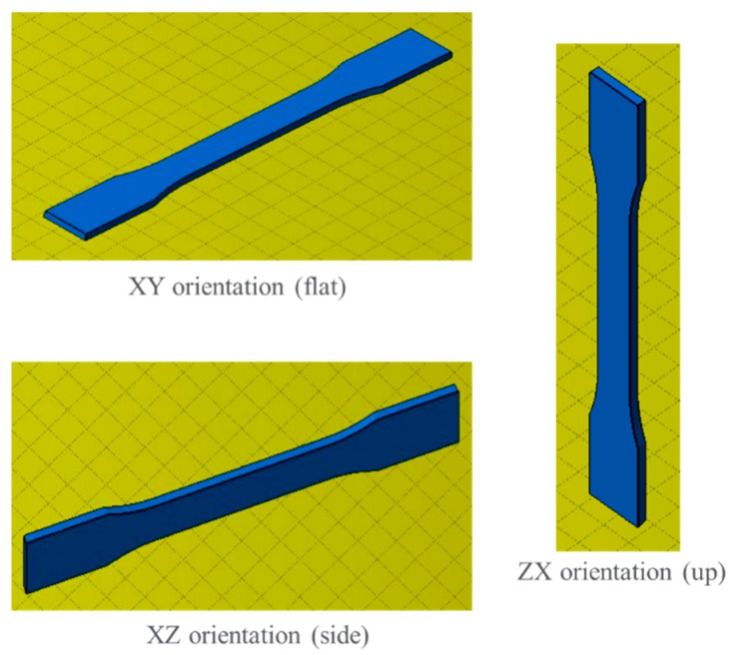
Printing orientation of tensile bars.

Many papers and data sheets of laser sintered/melted plastics only report the mechanical properties based on *xy* direction builds of tensile bars (see Figure 2) [15]. Indeed, in our previous works on PET [4,9], we had only obtained the *xy* orientation mechanical properties at that time. However, for a complete acceptance of a new material, it is necessary to provide the mechanical properties of *xy*, *xz*, and *zx* build orientations. End users are particularly concerned about *zx* orientation strength and elongation-to-break. Thus, we endeavored to give a more detailed mechanical characterization than we were able to do previously of PET articles printed from the PET powder. Table 4 shows our measurements of the mechanical data for PET and PA12, for *xy*, *xz,* and *zx* build orientations, measured with PET powder T1, printed under optimized conditions in the laboratory machine. The orientations of the tensile bars during the building of the bars are shown in Figure 2.

Table 4 shows the tensile and flexural modulus of PET are almost double those of PA12. This result is to be expected from PET’s higher *T*_g_ (78 °C for PET versus ≈40–50 °C for PA12). Also noteworthy is that in PET, the modulus and the strengths for *xy*, *xz* and *zx* build orientations are closer to each other—that is, there is near-isotropy in these properties.

The elongation-to-break is held as a crucial factor in the introduction of a new material. PA12 generally has an elongation-to-break value of ≈13% for *xy* orientation; in injection molded PA12, this is about 200%. However, in the *zx* orientation, elongation-to-break was 2.6% for PA12, from our measurements (Table 4). The bars from PET powder T1 printed in the laboratory machine showed lower values (4.9% and 5.0% in the *xy* and *xz* directions respectively) and 2.1% in the *zx* direction. Often end users demand new materials to show the same elongation-to-break as PA12. But it is not possible to achieve high elongation-to-break with a higher *T*_g_ polymer with higher modulus and strength. The other factor that determines the elongation-to-break in the laser melted part is its void content. The only way to improve the elongation-to-break for a high *T*_g_ material is to reduce the void content. In the current PET parts, the void content was ≈2%; lowering this content in the future work would increase the elongation-to-break. Parts from PET must be designed with these factors in mind.

**Figure 3:**
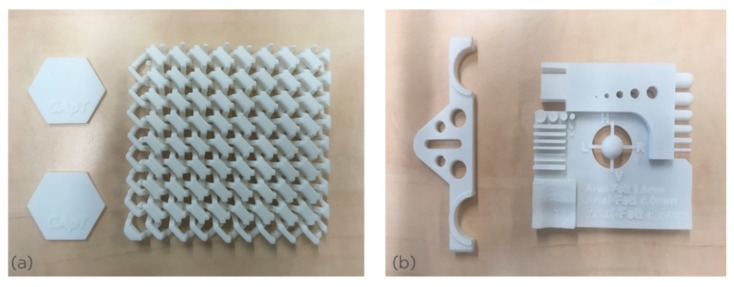
(**a**) Hexagonal plates with lettering (GApT), and chain mail, printed from PET powder T1 in the laboratory machine. (**b**) Bracket (**left**) and dimension test bar (**right**) printed from PET powder T1 in the laboratory machine. The parts are uniformly semi-crystalline.

Figure 3 shows complex parts can be built from PET powder T1 in the laboratory machine. The density of the article printed from powder T1 was around 1.37 g/cm^3^ after allowing for the 2% porosity translates to a crystallinity of 30%. Figure 1c shows the DSC curve of a printed tensile bar. This is the characteristic DSC curve of a semi-crystalline PET with heat of fusion of 44.8 J/g. It confirms that the process leads to the transition “crystalline PET powder to crystalline PET article.” This is not usually the case with injection molding of PET where the transition is most often “crystalline pellets to amorphous article,” if cold molds are used.

### 3.3. Printing of PET Powder T2 in the Laboratory Machine

One major difference between powder T2 and T1 is the I.V. (0.84 dL/g versus 1.122 dL/g for powder T1).

**Figure 4:**
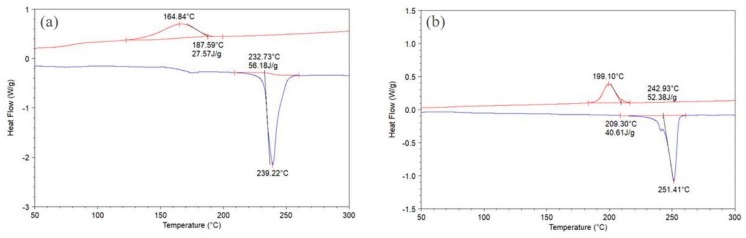
(**a**) DSC of PET powder T2. The melting peak is at 239 °C with heat of fusion of 56.2 J/g; (**b**) DSC of PET bar printed from T2 powder in the laboratory machine. The melting peak is at 251 °C and the heat of fusion is 40.6 J/g.

Figure 4a shows the DSC curves of PET powder T2. The process window for this powder was equal to melting onset T − crystallization onset T: 233 − 188 = 45 °C (see Table 3).

**Figure 5:**
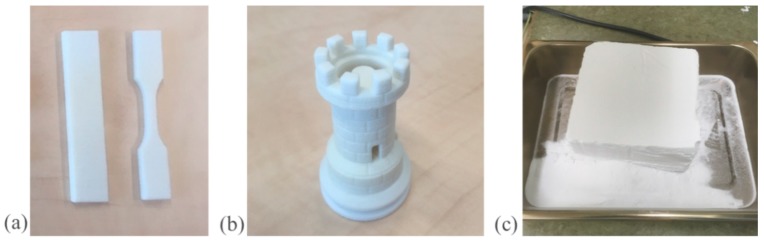
(**a**) The *zx* orientation bars printed from PET powder T2. (**b**) Complex part printed from PET powder T2. (**c**) The PET powder T2 cake when using the build bed at 228 °C was a little hard.

With the laboratory machine, we applied similar processing parameters to PET powder T2 as used with PET powder T1 (Table 2). The powder laying process from the reservoir to the build table was very good. In the part building stage, warping curling and cracking were not observed. Figure 5a shows printed bars and Figure 5b shows a more complex part (a chess rook) printed from PET T2 in the laboratory machine. The part definition was very good.

Initially, we tried PET powder T2 with the same processing parameters as used for PET powder T1 in Table 1; that is, the bed was kept at 228 °C. While the printed parts were fine, we observed the powder cake left behind was a little too hard compared with the PA12 powder cake (see Figure 5c). The hardness of the powder cake is one of the important characteristics that needs to be monitored for the powder. The cake should not be very hard; otherwise, the parts cannot be cleaned easily. A hard cake could also mean that the parts are out of control on the dimensions. Hence, in a second printing episode we used 225 °C for the build bed (as in Table 2), and the powder cake became softer and comparable with PA12. Powders T1 and T2 have comparable crystallinity (heat of fusion) but the lower I.V. of powder T2 compared with powder T1 (0.84 dL/g versus 1.122 dL/g) led to its self-sintering at a lower temperature. In self-sintering, the particles sinter together due to the heat and the pressure from the weight of the powder on top. An even lower print table temperature might be desirable for powder T2, but lowering it too much would lead to curl. Other than lowering the build bed temperature, no further optimizations were undertaken with powder T2 in the laboratory machine.

The mechanical properties of tensile bars printed from the powder T2 in the laboratory machine are shown in Table 5. It can be seen that the low strain properties such as the tensile and flexural modulus are high and similar to the bars from the high molecular weight PET powder T1 (Table 4); that is, the lower I.V. of the starting powder did not decreased these. However, there was a greater anisotropy with powder T2 printed in the laboratory machine, for the strengths (tensile and flexural) and the elongation-to-break, as the *zx* printed bar had significantly lower values for these than the *xy* and *xz* printed bars (Table 5 compared with Table 4). Only optimization of the bed temperature was done to avoid curl and caking of the powder bed, but no work has been done yet to optimize the process parameters with powder T2 as with powder T1, to obtain the best mechanical properties.

With PA12, Vasquez et al. [13] noted that the energy density, which is controlled by the combination of the laser power, the distance between scan lines, and the laser′s traverse speed, has a very strong influence on the density and the mechanical behavior of the parts. Hence, they need to be optimized. Caulfield et al. [16] have shown for PA12, the consolidation and extension-to-break increase with increasing energy density up to a certain point, but decline thereafter. Vasquez et al. [13] point out that too high an energy density can even lead to local vaporization, and the gases generated would add to the voids, and this would cause the decrease in mechanical properties.

The I.V. of the bars printed in the laboratory machine from PET powder T2 somewhat surprisingly showed an increase in I.V. from 0.845 to 0.877 dL/g. That is, it had gone above the value of the powder with an I.V. lift of 0.032 dL/g. In our previous works, the I.V. of the part always showed a small decrease relative to the powder.

Figure 4b shows the DSC of the bar from powder T2 printed in the laboratory machine. This also confirms that again the transition was “crystalline PET powder to crystalline PET part.” The heat of fusion of the part was lower than the starting powder T2 (40. 6 J/g versus 56.2 J/g) indicating that the crystallinity was a little less than the original powder. In fact, the decrease in crystallinity proves the process involves melting instead of sintering. Sintering will lead to increase in crystallinity (and heat of fusion), due to annealing, and this is shown in the work of Ahmad and Bashir on powder compaction of PET, which is based on true sintering with pressure [17]. Melting and re-crystallization can lead to an increase in heat of fusion, but a decrease in heat of fusion in the article can only occur if the powder had been melted.

### 3.4. PET Powder T2 Printed in the Commercial Machine

PET powder T2 was additionally printed in a commercial machine owned by LSS Laser-Sinter-Service GmbH, Germany. Most importantly, this machine could apply a laser scan speed of 12.7 m/s instead of 5 m/s, which is the normal currently with production machines. It was important to know if the PET would still print at production speed without distortions and allow complex parts to be built. These factors would test the versatility of the PET powder further and give it credibility as a new material for powder bed fusion.

Compared with the laser power we used for powder T2 with the laboratory machine, the laser power using the commercial machine was much higher (52 W) but with higher scan speed of 12.7 m/s and also higher scan spacing (Table 2).

**Figure 6:**
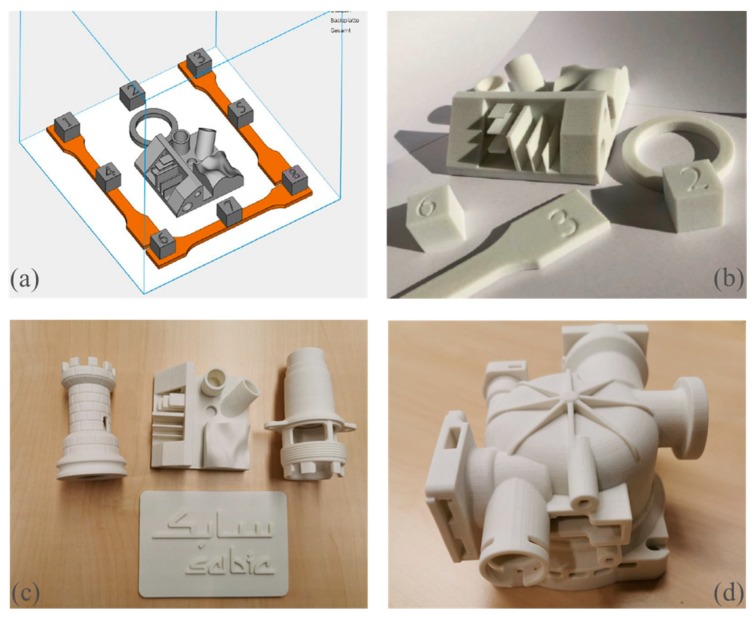
(**a**) Schematic of parts to test the capability of PET powder for complex shapes; (**b**) actual PET parts corresponding to diagram (**a**). (**c**) A chess rook, a part with freeform surface, a threaded part for an appliance, and a plaque showing accuracy of text in relief. (**d**) Complex design. These parts were printed from PET powder T2 in a commercial machine.

Figure 6a shows a diagram of a range of parts that was built in a single build. Figure 6b–d shows the actual parts. The selection of these parts was designed to examine (1) warpage and curl which can be easily visualized with the tensile bars, (2) sharpness of the contours and surface roughness which can be gauged with the cubes, (3) round geometry for visual inspection of the offsets, and (4) test geometry of overhanging angles (surface quality of down/up-facing surfaces, minimum wall thickness, and geometrical accuracy of the holes).

Unlike the experiments with the laboratory machine, the powder T2 was not pre-dried. The powder transfer was smooth although no flow promoter was added. The printing process with the parameters of Table 2 was stable; no curling occurred during the build process, there were no burnt areas, and neither dust nor smoke were not generated due to the high laser power.

After extraction of the samples (Figure 6b), there was very little post curling of the tensile bars; the freeform (curved) areas had a smooth surface; the contour sharpness of the round part (ring), and of the cubes, was good; the minimum vertical and horizontal wall thicknesses of 0.6 mm were built properly; and the overhanging angles had a smooth down-facing surface. Other complex shapes such as in Figure 6c,d were also built without difficulty. Some of these cannot be built as a single piece by injection molding. The color of the parts was beige instead of white, as seen with the articles made with the same powder using lower power and scanning speed in the laboratory machine (Figure 6b–d, versus Figure 5a,b). This would indicate that the energy density applied was at the upper limit where there is onset of degradation. As mentioned, the optimization of the applied energy has not been done yet for the commercial machine.

The DSC melting curve of a printed part using PET powder T2 from the commercial machine (not shown) again confirmed the transition during powder bed fusion was "crystalline PET powder to crystalline PET article.” However, this time the heat of fusion was 63 J/g, which was higher than the starting powder (56.2 J/g). Recall that the bars printed in the laboratory machine from the same powder T2 gave a melting peak of 251 °C and a heat of fusion of 40.6 J/g (Figure 4b), which was lower than that of the starting powder (56.2 J/g Figure 4a). In PET powder T1 also, and in our previous works with high I.V. PET powder, the printed PET parts were indeed crystalline, but the heat of fusion was typically 30–45 J/g; that is, generally lower than the starting powder [4,9].

The melting peak of the part printed in the commercial machine was also higher than in the starting powder T2 (melting peak of 251 °C, versus 239 °C for the powder in Figure 4a). The melting point of the surface is always lower than the bulk or interior of the particle. The powder has a larger surface area, so it has a depressed melting point compared with a pellet or an article. After powder bed fusion, the powder surface boundaries were removed in the article, so the melting point goes higher. A similar but smaller effect was observed with the high I.V. powder T1 and the part built from it in the laboratory machine (the melting peak was at 242 °C in the powder and 244 °C in the bar, see Figure 1a,c). The increase in crystalline perfection and crystallinity (higher melting point and higher heat of fusion) in the part over the powder is more apparent with the lower I.V. powder T2 than with powder T1. The higher heat of fusion of the printed parts from powder T2 in the commercial machine, compared to this powder (63 J/g versus 56.2 J/g) could be because the lower I.V. material crystallizes more perfectly from the melt and the annealing time (hours the part was left in the powder bed) was longer in the commercial machine than with the laboratory machine.

We can say that for PET, while powder bed fusion always creates the transformation “crystalline powder to crystalline part,” the degree of crystallinity in the parts, and hence, the melting point and heat of fusion of the parts can vary, as they depend on the cooling time in the bed. Our first work [4] achieved crystallinities <20% in the printed PET parts, which increased in the second work [9] to ≈30%, and in this work, with powder T2 and the commercial machine, the crystallinity of the article was actually higher than the starting powder. Kruth et al. [18] explained a “sintered” area remains molten until the part bed begins to cool. The cooling rate of the melt is very slow (hours) instead of seconds as in injection molding. Generally, the chains in a low I.V. melt are more mobile than in a high I.V. melt, and hence, can crystallize better. Thus, this work suggests that the PET part′s crystallinity depends on the I.V. and the thermal history of the molten part in the bed (build time and cool down time), and this has to be standardized if uniformity of crystallinity is desired in the article. 

The I.V. of the tensile bar made from the T2 powder in the commercial machine was 0.765 dL/g; that is, there was an I.V. drop of 0.845 − 0.765 = 0.080 dL/g. Although a PET with an I.V. of 0.765 dL/g is still very good for an injection molded article, when porosity is present, a large I.V. drop is very undesirable. A drop in I.V. after melting PET is normally expected, but it is opposite to what was observed with powder T2 in the laboratory machine, where we saw, surprisingly, an I.V. increase in the part. The reason for the large decrease in the part′s I.V. in the commercial machine for the same powder could be because too high of an energy density was used and the fact that the PET T2 powder was not pre-dried. We are investigating this further. Our previous results indicated the parts′ I.V. can be moderately lower than the starting powder [4,9]; here we found the parts′ I.V. can be substantially lower (as with powder T2 in the commercial machine), or even higher than the original powder (as with powder T2 in the laboratory machine). Clearly, the processing conditions affect the I.V. of the article. As for the I.V. of the residual powder T2 left in the build bed after the printing trials (≈12 h at 225 °C), it was 0.846 dL/g—compared with 0.845 dL/g for the original powder, and 0.765 dL/g in the printed part. That is, the powder was virtually unchanged. Gu et al. [9] had shown in a simulated heat-exposure study that the high I.V. PET powder (the same as the powder T1 here), did not show any great increase in I.V. after ageing for ≈100 h, and printed well even without addition of virgin powder [8]. This is a great feature of PET. In a printing episode, only 10%–20% of the powder in the build bed might be used. The unused powder stays in the heated chamber for hours, providing the support to the molten parts. With PA12, Gu et al. [9], and Dotchev and Yusoff [19], reported a 1.75–3 times rise in MW in the powder left in the build bed after a printing episode. Dotchev and Yusoff reported the re-use of such PA12 gave printing defects. As the powder is too expensive to discard, it is mixed with virgin powder in some proportion, depending on the polymer. Choren et al. [20], however, showed the prolonged use of recycled powder results in fluctuations in the mechanical properties.

Due to the high energy density used in the commercial machine, the mechanical properties of bars printed from the T2 powder were not the optimum (see Table 6). Like the results of the powder T2 run in the laboratory machine, the bars made in the commercial machine showed high values for the low strain properties, such as tensile modulus, but the strengths were lower and the elongation-to-break had dropped to under 2% (see Table 6). We are undertaking an optimization of the energy density (power, the scan speed, and hatch distance) with powder T2 in the commercial machine to improve the tensile strength and increase the elongation-to-break, which we know is possible, as shown with powder T1.

**Figure 7:**
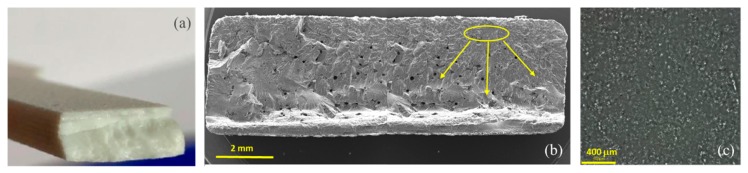
(**a**) Fractured tensile bar of PET powder T2 printed in the commercial machine. (**b**) SEM of cross section of the fractured tensile bar in (**a**) (left and right side of cross section of bar). Arrows indicate the crack initiation and growth regions. (**c**) SEM of surface of the tensile bar made from the powder T2 in the commercial machine, shows a significant amount of unmelts (coring).

The porosity of the cross section of a broken tensile bar made from powder T2 in the commercial machine was checked. The porosity was not high enough to be seen by eye (Figure 7a). The fracture surface did not show mm sized pores, visible by eye. The fracture surface was checked in the SEM and is shown in Figure 7b. There were globular internal voids towards the center of the cross section of the bar with size range 50–200 µm. The porosity was at a similar level as in our previous work corresponding to a void level of ≈2%. This void level is similar to what is obtained with a good plastic powder, such as PA12. The main crack during the tensile test appears to have been initiated from the top (that is, the bar’s surface) right edge (see Figure 7b). Secondary cracks initiated from the internal voids.

As the cracks appeared to have initiated from the bar’s principal surface, this was examined in the SEM. Figure 7c shows a picture of the bar’s surface. This surface had some fine embedded particles with size range 1–10 µm. The presence of unmelted particles, as in Figure 7c, is referred to as coring; it was not seen in our previous works [4,9]. In PA12, coring has been noted by Majewski et al. [21] as well as Zarringhalam [22]. They proposed a concept of a “degree of particle melt” to characterize parts printed from PA12. Larger particles require more power to fuse them, and a large variation of particle size can lead to melting of the finer particles and incomplete melting of the bigger particles, and this finally has a bearing on mechanical properties [23]. This would not occur in injection molding. However, such issues can often be solved, by increasing the laser power for the outline scan, reducing the scanning speed, and sometimes by multiple scans. As the fracture cross-section in Figure 7b suggests the cracks started at the surface and moved inwards due to these inclusions, the remedy would be to increase the energy density of the outline scan.

### 3.5. PET as a Material for Powder Bed Fusion, versus Injection Moulding

Injection molding can create amorphous PET parts that are not feasible by powder bed fusion; on the other hand, the latter can create crystalline parts from PET that are not possible through injection molding. We would like to explain why.

In a patent application, Bashir and Gu claimed polymer compositions with a crystallization half time of between 30 s and 12 min at a supercooling of 50 °C below *T*_m_ are suitable for 3D printing via powder bed fusion [4]. Besides PET, this includes poly(trimethylene terephthalate), poly(ethylene napthalate), poly(ethylene furanaote), poly(hydroxy butyrate), and poly(ethylene succinate). Example of polymers with shorter crystallization half times are polyethylene (PE), polyoxymethylene (POM), polypropylene (PP), and polybutylene terephthalate (PBT). These plastics would be more difficult to use than PET for powder bed fusion as the crystallization would be too fast, and that would cause warpage. In the DSC, the melting and crystallization peaks are closer with these materials. On the other hand, powders of crystallizable polymers with too long a crystallization half time would not form crystalline articles, but would yield amorphous ones. Crystallizable polymers with an intermediate crystallization half times like PET would transform from crystalline powders to melts after laser exposure, and the melt crystallizes slowly as it descends into the powder bed. Thus, Figure 6b shows both simple and complex PET parts made by powder bed fusion are always semi-crystalline, in both thin and thick sections. 

**Figure 8:**
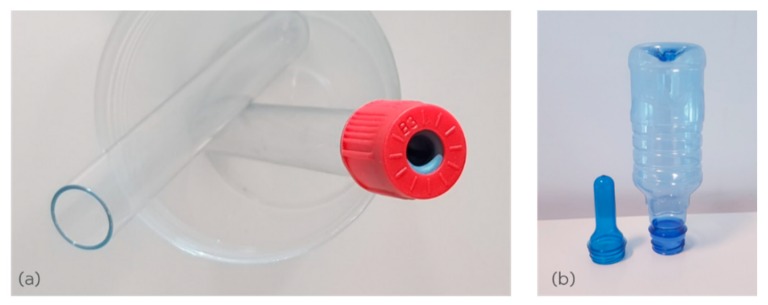
(**a**) Thin walled injection molded, amorphous PET blood tube. The wall thickness is <1 mm, and hence, the tube is rapidly quenched and is uniformly amorphous. An amorphous PET part like this cannot be made by powder bed fusion. (**b**) Thin walled injection molded, amorphous PET preforms (left) are converted to semi-crystalline PET articles (biaxially-oriented bottles, right). Amorphous PET parts for post-stretching cannot be made by laser melting of PET powder.

As the crystallization rate of PET is slow (relative to PBT, POM, PE, polypropylene, and PA6), it is possible to obtain it in both the amorphous or semi-crystalline states—at least in injection molding. The amorphous state is obtained by quench-cooling molten PET, while the semi-crystalline state is obtained by slow cooling of the melt. Thus, the injection molded PET articles, if thin walled will be amorphous and transparent, but if thick walled, will be inhomogeneous (amorphous at the exterior and crystallized in the interior). Even if the PET article is homogeneously amorphous, the *T*_g_ of the PET is 78 °C; hence, amorphous PET articles would have limited end use, as they would be confined to be employed essentially at room temperature. A rare use of amorphous PET in an end-use article is injection molded vacuum blood tubes; these are employed as attachments to syringes to suck blood for medical testing (Figure 8a). These transparent PET tubes have a wall thicknesses <1 mm, and after vacuum evacuation are sealed with a cap containing a pierceable rubber septum; the PET blood tubes can be used in the amorphous state, as the application does not cross room temperature. While it is often claimed that additive manufacturing allows parts to be built that cannot be made by injection molding at least in terms of complexity, it is worth pointing out here that such transparent, amorphous PET parts as in Figure 8 cannot be made at all by powder bed fusion, because the process does not allow quick quenching from the melt, but leads to slow thermal crystallization in the hot build bed.

Transparent, amorphous preforms of PET with wall thickness of ≈1–3 mm that are made via injection molding have another useful property. They can be reheated above the *T*_g_ and stretched and crystallized. In this case, the molded amorphous preforms are intermediates for the production of semi-crystalline, biaxially-oriented bottles by a stretching process (Figure 8b). In such end uses, the PET is used in a stretched form; this increases the strength greatly and reduces the amount of material that has to be used. Again, powder bed fusion cannot be used to make amorphous PET preforms for hot post stretching above the *T*_g_, for two reasons: the printed PET part would always crystallize and there would be about 2% void content; both factors will prevent the stretch ability.

### 3.6. PET Versus other Thermoplastics for Powder Bed Fusion

There are many publications on PA12, and quite a few on PEEK, PP, polyoxymethylene (POM), PBT, and PA6 powders for powder bed fusion [24]. Further, these materials are commercially available, with PA12 leading. Apart from our previous papers [4,9] and patent applications [5], there are no other publications on PET powder, and hence the question will arise where PET will be positioned amongst the materials for powder bed fusion.

We may divide the available crystalline thermoplastics into those fusing below 200 °C, those fusing above 200 °C but below 300 °C, and those melting above 300 °C. PA12, PA 11, PP, and POM fall in the first category. PEEK falls above 300 °C; it is the most expensive and difficult to process. PET falls in the second category, along with PA6 and PBT. The melting point of PET is ≈250 °C, while PA6′s and PBT′s are around 220 °C.

Hence, we needed to assess PET′s performance in powder bed fusion versus PA6 and PBT. The PA6 is more prone to discoloration in the left-over powder due to the heat, but it can be solved partly by using a black instead of white PA6 powder. A more serious problem with PA6 is its moisture pick up is relatively high around 3% by weight). This means printed articles have to be conditioned in an atmosphere with controlled humidity. However, if the conditioned PA6 article is moved to a drier region, it would dry out, contract, and become brittle. Amorphous PET′s maximum moisture up take is 0.4% by weight, while semi-crystalline PET has a maximum uptake of 0.2%. The PET articles from powder bed fusion are crystalline, and hence the moisture uptake will be 0.2% or less. Thus, they would not need to be conditioned in a controlled atmosphere, and there is no problem of dimensional change with environment. This is clearly advantageous over PA6.

It is when thick, uniformly crystalline unoriented articles are required by injection molding, PET has limitations, unless hot molds are used. Thus, PET is not considered a good injection molding thermoplastic. PBT is the polyester par excellence for injection molding. We observed, however, that the roles are reversed in powder bed fusion: PET will always be better than PBT. While PET is not competitive with PBT in injection molding due to its slower crystallization, this very trait of PET makes it particularly suitable for producing uniformly crystalline articles by powder bed fusion, and conversely, the fast crystallization of PBT that makes it preferred for injection molding, poses problems for controlling curl in powder bed fusion of PBT.

Unlike injection molding which operates on short cycle times of seconds or tens of seconds, the powder bed fusion process has inherently long cycle times of hours, and it leaves the article in the powder bed to crystallize slowly. In this case, the powder bed fusion process delivers articles from PET that cannot be made by injection molding, not only in the sense of part complexity, but in terms of crystallinity. In unoriented parts for end use, crystalline articles would be preferred over amorphous ones because the thermomechanical properties will not be dominated by the *T*_g_ but by the *T*_m_. In PET, the *T*_g_ is 78 °C and the *T*_m_ is 256 °C, compared with a *T*_g_ of ≈55 °C and a *T*_m_ of 223 °C in PBT. Crystalline PET has a somewhat higher modulus and strength than PBT, but PBT has the higher impact toughness. The intrinsic polymer cost for PET is lower than PBT.

Wegner et al. [25] reported powder bed fusion of PBT powders led to high modulus of up to 3000 MPa, but the strength values were below 40–45 MPa and the elongation-to-break was 1%–2%. Kleijnen et al. [26] made a PBT powder for powder bed fusion that had a processing window of only 7.6 °C, and printing simple bars was difficult enough, due to curl. The bars that could be printed showed a modulus of 2211 MPa, a strength of 20.3 MPa, and an elongation-to-break of 1%. The properties of the printed PBT available from these works are collected together in Table 7, and contrasted with PET. Even without any optimization, PET gives better values. Table 7 also compares the mechanical properties of parts made by powder bed fusion and injection molding. The injection molded PET we used in Table 7 had the same I.V. as the PET powder T2 used here for powder bed fusion (0.84 dL/g).

PET is a plastic that can truly be used in a circular economy. The recyclability of PET arises from the fact that its molecular weight can be rebuilt by melt or solid-state polycondensation. The absence of cross-linking in PET after thermal exposure in the printer also means any unused powder can be converted to pellets as well, for conventional extrusion processing. Likewise, the printed articles of PET can also be ground, and re-used for melt extrusion or for conversion back to powder for powder bed fusion.

## 4. Conclusions

In this work, we have shown the full *xy*, *xz,* and *zx* mechanical data to make a convincing case for PET as a good powder for powder bed fusion, occupying a material space in the 200–300 °C bracket.

The modulus of printed PET was almost double that of PA12. Further, with PET, if the laser processing conditions are optimized, there is not much difference in *xy*, *xz,* and *zx* orientation moduli and strengths (that is, near isotropy can be obtained). Usually, the *zx* orientation is significantly lower than the other two. The elongation-to-break was lower than for PA12, but it was not in an unusable range. The density of the PET is higher than PP and PA12.

The intrinsic properties of PET powder (melting width and process window) are suitable for powder bed fusion. The PET powder was also process tolerant. High I.V. and lower I.V. PET powders were equally easy to print into complex shapes. Annealing effects in the part are more apparent with lower I.V. PET. Curl was easy to control. The surfaces of the printed free-forms were smooth, and sharp contours were obtainable. Vertical and horizontal walls of thickness of 0.6 mm were built properly and the overhanging angles had a smooth down facing surface.

It is often pointed out that powder bed fusion allows complex parts such as lattice structures to be made which cannot be made via injection molding. For PET specifically, there is another benefit that powder bed fusion can deliver which is not easily attained by injection molding. Normally, crystalline articles of PET are realizable only after a stretching process (fibers, films, bottles). It is difficult to make crystalline articles from PET via injection molding due to the polymer’s slow crystallization, and for this reason, PBT is the preferred polyester over PET for injection molding. However, this very feature of PET (slow crystallization) makes it particularly good for powder bed fusion. It renders a wide process window to avoid curl and lateral spread, and it leads to uniformly crystalline PET articles due to the annealing effect of the hot powder bed.

The PET powder will be positioned in the space of PA6 and PBT. Due to the low moisture uptake, lower discoloration and high melting point, it will be superior to PA6. Due to the slow crystallization, the process window is wider for PET than for PBT, and it is easier to control curl. The recyclability of the PET is also very good. 

At the same time, it has to be declared that powder bed fusion cannot make amorphous PET articles and preforms for post-stretching. Although the laser melts the PET, the melt cannot be quench-cooled to the amorphous state. Amorphous articles and preforms can be made from PET only by injection molding. Hence, for PET the powder bed fusion process can be considered to be a complementary fabrication technology to injection molding, rather than a replacement.

## Figures and Tables

**Table 1:** Processing parameters for PET powder T1 and PA12 in the laboratory machine.

Parameters		PA12	PET T1
Machine		The laboratory machine	
Temperature	Build area temperature (°C)	166	228
	Feed temperature (°C)	120	160
Laser	Scan count	Single	Single
	Power (W)	17–19	20–30
	Scan speed (m/s)	5	5
	Scan spacing (µm)	100	100
Other	Layer thickness (µm)	120	100

**Table 2:** Selective laser melting parameters for PET powder T2 in both laboratory and commercial machines.

Parameters		PET Powder T2	
Machine		Lab.	Commercial
Temperature	Build area temperature (°C)	225	233
	Feed temperature (°C)	160	160
Laser	Scan count	Single	Single
	Power (W)	22–25	52
	Scan speed (m/s)	5	12.7
	Scan spacing (µm)	100	200
Other	Layer thickness (µm)	100	100

**Table 3:** The melting width, heats of fusion, and processing windows of PET T1 and T2, and PA12, from their DSC curves.

Powder	Melting Width of DSC Peak (°C)	Heat of Fusion (J/g)	Processing Window ^1^ (°C)
PET T1	32	56.1	52
PET T2	40	56.2	45
PA12	19	110.2	37

^1^ Processing window = melting onset—crystallization onset in °C.

**Table 4:** Comparison of mechanical properties of bars printed from PET powder T1 with PA12, for different printing orientations. The bars were printed in the laboratory machine.

Property	Orientation	PA12	PET T1
Density (g/cm^3^)	*xy*	0.99	1.36
	*xz*	1.00	1.35
	*zx*	0.98	1.35
Tensile Modulus (MPa)	*xy*	1596	2961
	*xz*	1486	3093
	*zx*	1443	2968
Tensile Strength (MPa)	*xy*	43	66
	*xz*	43	66
	*zx*	33	64
Elongation at Break (%)	*xy*	13.1	4.9
	*xz*	11.0	5.0
	*zx*	2.6	2.1
Flexural Modulus (MPa)	*xy*	1685	2713
	*xz*	1702	2464
	*zx*	1177	2607
Flexural Strength (MPa)	*xy*	68	113
	*xz*	47	103
	*zx*	58	80

**Table 5:** Mechanical properties of PET articles made in the laboratory machine from powder T2. The process parameters are shown in Table 2. Note the scanning speed is 5 m/s for the laboratory machine.

Property	Orientation	PET T2
Tensile Modulus (MPa)	*xy*	3260
	*xz*	3330
	*zx*	2998
Tensile Strength (MPa)	*xy*	64
	*xz*	64
	*zx*	37
Elongation at Break (%)	*xy*	2.7
	*xz*	2.7
	*zx*	1.4
Flexural Modulus (MPa)	*xy*	3103
	*xz*	2954
	*zx*	2767
Flexural Strength (MPa)	*xy*	95
	*xz*	92
	*zx*	59

**Table 6:** Mechanical properties of PET articles made in the commercial machine from powder T2, without optimization. The processing parameters are in Table 2, and the scanning speed was 12.7 m/s.

Property	Orientation	PET T2
Tensile Modulus (MPa)	*xy*	3388
	*xz*	3492
	*zx*	3325
Tensile Strength (MPa)	*xy*	49
	*xz*	50
	*zx*	37
Elongation at Break (%)	*xy*	1.5
	*xz*	1.5
	*zx*	1.1

**Table 7:** Mechanical properties of PET and PBT articles made by powder bed fusion (PBF) ^1^ and injection molding (IM).

Property	Amorphous PET	Crystallized PET	Crystallized PET	PBT	PBT	PBT
Processing	IM	IM	PBF	IM	PBF	PBF
Tensile Modulus (GPa)	1.6	1.6	3.0	2.3	2.2	2.6–3.08
Tensile Strength (MPa)	60	63	66	53	20	32–40
Elongation at Break (%)	96	5.2	4.9	112	1	1–2.2
Flexural Modulus (MPa)	2.5	3.5	2.7	n/a	n/a	n/a
Flexural Strength (MPa)	89	132	113	n/a	n/a	n/a
Remarks			Powder T1	[24]	[26]	[24]

^1^ For PBF parts, the printing orientation is *xy* direction.
